# Expression of PD-1 and PD-L1 in Extramammary Paget Disease: Implications for Immune-Targeted Therapy

**DOI:** 10.3390/cancers11060754

**Published:** 2019-05-29

**Authors:** Shakuntala H. Mauzo, Michael T. Tetzlaff, Denái R. Milton, Alan E. Siroy, Priyadharsini Nagarajan, Carlos A. Torres-Cabala, Doina Ivan, Jonathan L. Curry, Courtney W. Hudgens, Jennifer A. Wargo, Aysegul A. Sahin, Curtis A. Pettaway, Victor G. Prieto, Phyu P. Aung

**Affiliations:** 1 Department of Pathology, The University of Texas MD Anderson Cancer Center, Houston, TX 77030, USA; drmauzos@gmail.com (S.H.M.); mtetzlaff@mdanderson.org (M.T.T.); asiroy@ufl.edu (A.E.S.); pnagarajan@mdanderson.org (P.N.); ctcabala@mdanderson.org (C.A.T.-C.); dsivan@mdanderson.org (D.I.); jlcurry@mdanderson.org (J.L.C.); asahin@mdanderson.org (A.A.S.); vprieto@mdanderson.org (V.G.P.); 2 Department of Translational Molecular Pathology, The University of Texas MD Anderson Cancer Center, Houston, TX 77030, USA; cwhudgens@mdanderson.org; 3 Department of Biostatistics, The University of Texas MD Anderson Cancer Center, Houston, TX 77030, USA; drmilton@mdanderson.org; 4 Department of Surgical Oncology, The University of Texas MD Anderson Cancer Center, Houston, TX 77030, USA; jwargo@mdanderson.org; 5 Department of Urology, The University of Texas MD Anderson Cancer Center, Houston, TX 77030, USA; cpettawa@mdanderson.org

**Keywords:** extramammary Paget disease, mammary Paget disease, PD-1, PD-L1, immune infiltrate

## Abstract

Extramammary Paget disease (EMPD) is a locally aggressive cutaneous malignancy that usually arises in anogenital or axillary skin. Immune checkpoint inhibitors targeting programmed cell death receptor (PD-1) and/or its ligand (PD-L1) are approved for the treatment of several types of cancer, and response to these generally correlates with increased PD-L1 expression by tumor cells. The expression of PD-L1 and composition and density of the tumor-associated immune infiltrate in EMPD have been little studied. To determine whether EMPD might be amenable to immune checkpoint blockade, we analyzed the expression of PD-1 and PD-L1 and the composition and density of the tumor-associated immune infiltrate in EMPD and evaluated associations between biomarker expression and clinicopathologic parameters. Twenty-one EMPD tumors were evaluated for tumor cell PD-L1 expression and for relative expression and distribution of CD3, CD8, PD-1, and PD-L1 in the tumor-associated immune infiltrate by using a combination of visual and image analysis (Aperio ImageScope). In addition, PD-L1 expression was assessed in 10 cases of mammary Paget disease (MPD). In EMPD cases, PD-L1 was expressed by tumor cells (3/21; 14%) and the tumor-associated immune infiltrate (15/21; 71%), and PD-1 was expressed by the tumor-associated immune infiltrate in all cases analyzed (18/18). However, PD-L1 expression by EMPD tumor cells did not correlate with the density of CD3-, CD8-, or PD-1-positive cells in the tumor-associated immune infiltrate or other clinicopathologic parameters. Furthermore, the density of CD3, CD8, PD-1, and PD-L1 in the tumor-associated immune infiltrate did not correlate with any clinicopathologic parameters evaluated with the exception that CD3 positive values were significantly higher in patients who were still alive (median, 1310 cells/mm^2^; range, 543–2115;) than in those who died (median, 611 cells/mm^2^; range, 481–908; *p* = 0.049). In all MPD cases, PD-L1 was absent in tumor cells but present in the tumor-associated immune infiltrate, and PD-L1 expression in lymphocytes was lower in patients with HER2/neu-positive than in those with HER2/neu-negative disease (*p* = 0.07). Our findings raise the possibility of therapeutic targeting of the PD-1/PD-L1 axis in EMPD.

## 1. Introduction

**Figure 1:**
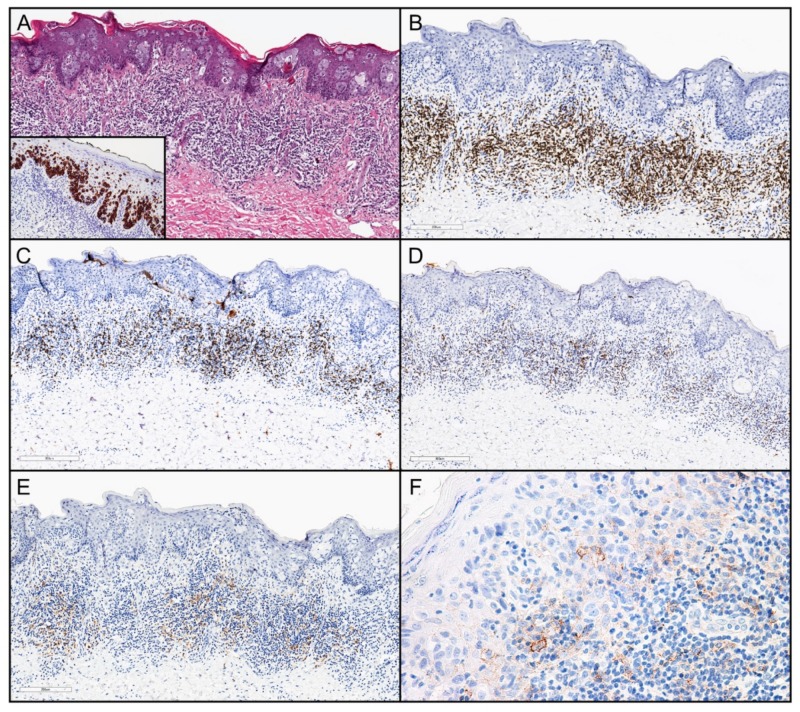
Immune infiltrate associated with extramammary Paget disease (EMPD). (**A**–**E**) Representative hematoxylin-eosin-stained sections showing (**A**) intraepithelial tumor component (100×) (inset, cytokeratin-7 immunohistochemical (IHC) study highlighting EMPD tumor cells; 100×) and (**B**) CD3+ lymphocytes (100×), (**C**) CD8+ lymphocytes (100×), (**D**) PD-1+ lymphocytes (100×), and (**E**) PD-L1+ lymphocytes (100×) in the tumor-associated immune infiltrate. (**F**) Weak and predominantly partial membranous expression of PD-L1 in EMPD tumor cells (200×), shown for comparison (1–2+ intensity).

Extramammary Paget disease (EMPD) is an uncommon cutaneous adenocarcinoma most often arising in older patients (approximately 95% of patients were >50 years old) and in the anogenital area (approximately 90% of cases) or axilla (approximately 7% of cases) [1,2,3,4]. The tumor cells are most often confined to the epithelium (Figure 1A) but occasionally invade the underlying dermis or submucosa. Currently, treatment of EMPD generally includes aggressive surgical extirpation, but this can be associated with high patient morbidity [5,6]. Even with aggressive surgical excision, EMPD exhibits a high rate of local recurrence (44 of 174 cases (25%) in a Mayo Clinic study [7]) owing to multifocality and subclinical extension; rarely, nodal and systemic metastases develop (10 of 261 cases (3.8%) in the Mayo Clinic study [7]). Effective systemic therapies for metastatic or locally advanced disease are lacking.

Immune checkpoint inhibitors have recently been approved by the US Food and Drug Administration (FDA) for treatment of non-small cell lung cancer, melanoma, and other types of cancers [8,9,10,11,12]. Many immune checkpoint inhibitors target programmed cell death receptor (PD-1) and its ligand (PD-L1) interaction. Interaction between the PD-1 receptor on cytotoxic T cells and its ligands PD-L1 and PD-L2 on tumor cells is a mechanism by which neoplastic cells can dampen the tumor-specific immune response and evade anti-tumor immunity [13]. Immune cells in the tumor microenvironment, including T lymphocytes [14,15] and macrophages [16,17], are also known to express PD-L1, interact with PD-1 on cytotoxic T cells, and contribute to tumor immune escape. Thus, blocking immune checkpoints can enhance the anti-tumor responses by activation of the tumor-associated immune infiltrate and propagation of the anti-tumor immune response. Our previous study showed a direct relationship between the density of the tumor-associated immune infiltrate and/or tumor cell expression of PD-L1 with clinical response to immune checkpoint blockade [18].

Given the advances of immunotherapy in other tumors and the paucity of effective agents to treat locally advanced or metastatic EMPD, we sought to quantify the density and composition of the tumor-associated immune infiltrate in EMPD and further to determine PD-L1 expression in EMPD tumor cells.

## 2. Results

### 2.1. Patient Demographic and Clinical-Pathologic Characteristics

Demographic and clinical-pathologic characteristics for the 21 patients with EMPD are summarized in Table 1 and Table 2. Our cohort consisted of 11 men and 10 women with a mean age of 68 years (range, 48–79 years). Twenty specimens were from available primary tumors, and one was from a metastatic lymph node since the primary tumor tissue was not available for further study. Ten patients had a history of other cancers, and seven of these patients had another non-skin cancer involving the prostate in two patients, breast in two, colon–rectum in one, kidney (clear cell carcinoma) in one, and lung in one. The median follow-up period for all EMPD patients was 59.2 months (range, 2.8–185.0 months).

### 2.2. EMPD Treatment and Outcomes

All patients were initially treated with wide local excision, after which 17 patients (81%) had positive margins. Of the 17 patients with positive margins, nine underwent re-excision, and eight of these nine continued to have positive margins despite multiple attempts at surgical extirpation. One patient received laser ablation and fulguration for residual disease, and one patient received topical imiquimod treatment for recurrent disease.

Seven (33%) of the 21 patients experienced local recurrence, and one patient had persistent disease despite treatment. Three patients (14%) with metastatic disease received docetaxel and carboplatin. One of the three patients also received intensity-modulated radiation therapy to a total dose of 66 Gy in 33 fractions to the primary tumor site and bilateral inguinal lymph nodes. One patient underwent bilateral superficial and deep inguinal lymph node dissection and salvage systemic therapy with gemcitabine, 5-fluorouracil, cisplatin, and leucovorin for a total of seven cycles for recurrent tumor after initial chemotherapy. All three patients with nodal and systemic metastasis died of disease. None of the five patients with negative final margins after wide local excision experienced local recurrence or metastasis. 

One patient (patient 17 in Table 2) had a tumor broadly involving the anogenital area with extension to vulva, vagina, and ectocervix diagnosed on subsequent hysterectomy. Seven patients had an associated invasive component. One of these seven patients (patient 20 in Table 2) had a concurrent rectal adenocarcinoma, and the EMPD in this patient likely represented secondary EMPD (CK7+, CK20+, CDX2−). One patient (patient 17 in Table 2) had invasive adenocarcinoma involving the urinary bladder diagnosed 11 years after an initial diagnosis of extensive anogenital EMPD; this bladder adenocarcinoma was likely related to the EMPD, as suggested by its immunophenotype (CK7+, CK20−, GCDFP15+).

Of the seven patients with invasive EMPD, three (patients 15, 18, and 21 in Table 2) had the depth of invasion reported, as focal invasion, <0.5 mm and 10 mm, respectively. The patient with 10 mm invasion developed systemic metastasis, which was treated with chemotherapy, and died of disease. The patient with <0.5 mm invasion did not have nodal or systemic metastasis. She was treated with multiple local excisions, laser therapy, and imiquimod and was alive with multiple recurrences 14 years after initial diagnosis of EMPD and 13 years after diagnosis of invasive disease. The patient with focal invasion had wide local excision for EMPD with positive margins and was lost to follow-up.

### 2.3. Expression of PD-L1 in EMPD and MPD

A representative example of EMPD and the density and composition of the tumor-associated immune infiltrate is shown in Figure 1. Results of analysis of immunohistochemical (IHC) staining in the EMPD cases are summarized in Table 2. The expression of PD-L1 in tumor cells was detected in three of 21 (14%) EMPD cases but none of the MPD cases. In contrast, PD-L1 expression in the tumor-associated lymphocytic infiltrate was detected in 15 of 21 (71%) EMPD cases and all 10 (100%) MPD cases. Among the patients with mammary Paget disease (MPD), PD-L1 expression in lymphocytes was lower in patients with HER2/neu-positive disease (median H-score, 2.0; range, 1.0–6.0) than in patients with HER2/neu-negative disease (median H-score, 40.0; range, 6.0–60.0; *p* = 0.07). In the EMPD cases, none of the clinical-pathologic parameters assessed (including overall survival, disease-specific survival, and time to metastasis) or the relative density of CD3+, CD8+, or PD-1+ cells in tumor-associated lymphocytes quantified by automated image analysis (positive cells/mm^2^) significantly correlated with PD-L1 positivity (H-score) in tumor cells (Supplementary Materials Table S1).

### 2.4. Correlation of Density and Composition of Tumor-Associated Immune Infiltrates with Clinical-Pathologic Parameters

Immunohistochemical studies for CD3, CD8, and PD-1 were performed, and the relative densities of IHC+ cells associated with the tumor (Figure 1) were quantified using automated image analysis. Patients who were still alive at last follow-up had significantly higher CD3+ values (median, 1310 cells/mm^2^; range, 543–2115) compared with those who died (median, 611 cells/mm^2^; range, 481–908; *p* = 0.049). None of the other clinical-pathologic parameters assessed (including overall survival, disease-specific survival, and time to metastasis) significantly correlated with PD-L1 positivity (H-score) in tumor cells or with the relative density of CD3+, CD8+ or PD-1+ cells in tumor-associated lymphocytes (Table 2 and Supplementary Materials Tables S1–S4).

## 3. Discussion

In our study, PD-L1 was expressed in tumors in three of 21 EMPD cases and in the tumor-associated immune infiltrate in 15 of the 18 EMPD cases evaluated by automated image analysis. A prior study in metastatic bladder carcinoma showed that tumors with PD-L1-positive tumor-infiltrating immune infiltrates had higher response rates to anti–PD-L1 therapy [19]. Thus, our findings suggest that immune checkpoint blockade might be a feasible approach for locally advanced or metastatic EMPD.

The upregulation of PD-L1 in tumor cells has been identified in basal, ERBB2-enriched, and inflammatory breast cancers [20,21,22]. The upregulation PD-L1 also correlated with better response to neoadjuvant chemotherapy in basal and ERBB2-enriched breast cancers [20]. Currently, several clinical trials are evaluating the effectiveness of checkpoint inhibitors targeting PD-1/PD-L1 in breast cancer [23]. In this study, we found lower PD-L1 expression in lymphocytes of MPD patients with HER2/neu expression. However, additional studies with larger sample sizes are necessary to further evaluate these preliminary data.

In a previous study [24], PD-L1 was not expressed by any neoplastic cells of EMPD or MPD or the associated lymphocytes. In contrast, in our study, tumor cells did not express PD-L1 in any of the MPD cases, but tumor cells expressed PD-L1 in 14% of the EMPD cases. In addition, PD-L1 in the tumor-associated lymphocytic infiltrate was detected in 71% of the EMPD cases and all of the MPD cases. The discrepancy in findings between our study and the previous study might be due to the differences in dilutions or methods of using PD-L1 antibody despite the same clone (22C3) and similar cut-off values for interpretation. In that previous study, PD-L1 (Dako Agilent, clone 22C3, 1:50) was used with a cut-off value of 1% for positive PD-L1 expression. In our study, a commercially available FDA-approved PD-L1 antibody (pre-made kit) was used, and we followed the manufacturer’s recommendation for processing and interpretation [25]; the cut-off value for positive PD-L1 expression was 1%.

As expected with IHC assays, PD-L1 interpretation is not without challenges. Review of the literature shows different cut-off values for PD-L1 positivity, and different studies use different methods of PD-L1 scoring, basing it on tumor cells only, immune infiltrate only, or both [26,27,28,29,30,31]. There is poor interobserver agreement among pathologists in scoring PD-L1 in the tumor-associated immune infiltrate, and although patients with higher PD-L1 tumor proportion score might show clear benefit with immune checkpoint inhibitor treatment, subsets of patients with negative or lower levels of PD-L1 may also respond [32]. Hence, the FDA is currently not using cut-off values for PD-L1 by IHC in many tumors for which checkpoint inhibitors are approved. Although our study has certain limitations and only 14% of EMPD cases in our study had tumor cells positive for PD-L1, clinical trials of immune checkpoint inhibitors in EMPD might prove beneficial, especially in patients with metastatic EMPD. However, a larger study using similar analysis is necessary to confirm our findings.

In addition, our study showed that none of the clinical-pathologic parameters assessed (including overall survival, disease-specific survival, and time to metastasis) significantly correlated with the relative density of CD3+ cells in tumor-associated lymphocytes, with the exception that EMPD patients who were still alive had significantly higher CD3+ T cell densities than patients who died. Similarly, in the previous study mentioned above [24], the density of CD3+ T cells did not correlate with any of the clinical factors studied, including age, sex, localization, or recurrence. 

Moreover, our study shows that among the patients with MPD, PD-L1 expression in lymphocytes was lower in patients with HER2/neu-positive disease than in patients with HER2/neu-negative disease. However, the lack of available survival data and status of HER2/neu in MPD patients in the previous study [24] precludes a direct comparison of the findings and underscores the need for additional study with a larger sample size and complete clinical outcome data. 

There is a paucity of clinical studies examining therapeutic options for EMPD, and thus there is no widely accepted standard of care. The primary modality of treatment for EMPD is wide local excision or Mohs micrographic surgery [5,6]. Extramammary Paget disease is associated with a high rate of local recurrence after surgery [7], which is attributed to the difficulty of determining margin status due to the EMPD’s multifocal pattern of growth, tendency to arise as an ill-defined non-mass lesion, and frequent extensive subclinical extension. Non-surgical treatment options for EMPD include topical imiquimod [33], photodynamic therapy, radiation therapy, carbon dioxide therapy, anti-androgen therapy, targeted anti-HER2/neu therapy (trastuzumab) [34,35], and cytotoxic chemotherapy [7,36]. Currently, there is no widely accepted treatment regimen for metastatic EMPD, and case studies and reports have shown that the systemic chemotherapy-based regimens used in the past had limited success. Hence, it is necessary to explore the possible utility of immunotherapy for EMPD, at least for metastatic EMPD [37].

## 4. Materials and Methods

### 4.1. Case Selection and Diagnosis

With approval from the Institutional Review Board of the University of Texas MD Anderson Cancer Center (protocol no. PA16-0424), we identified and reviewed 21 cases of EMPD from 21 patients who were treated and followed at our institution from 2005 through 2016. Some of these cases may have been included in a previously published study [38]. Ten additional cases of MPD from 10 patients treated and followed at our institution from 2005 through 2016 were identified and reviewed. Patient records were reviewed to obtain demographic parameters (i.e., sex and age) and clinical-pathologic parameters (i.e., tumor anatomic site, final margin status of excision, associated invasive carcinoma, metastasis, local recurrence, history of other cancers, and vital status at last follow-up).

### 4.2. Immunohistochemistry

The protein expression of PD-L1 was assessed by immunohistochemistry using a commercial FDA-approved companion diagnostic assay (pre-made kit for PD-L1) in a CLIA-certified laboratory using the anti–PD-L1 antibody clone 22C3 (Dako, Carpentaria, CA), a mouse anti-human PD-L1 immunoglobulin G1-kappa generated through murine immunization with a fusion protein containing the human extracellular domain of PD-L1 [25]. In addition, IHC studies were performed using antibodies for CD3 (Dako A0452; 1:100), CD8 (LifeSciences Technology MS457s; 1:25), and PD-1 (Abcam ab137132; 1:250) on an automated Leica Bond immunostainer (Leica Biosystems, Buffalo Grove, IL, USA) and 3,30-diaminobenzidine chromogen per the manufacturer’s recommendations. We performed a positive control and a negative control in every run of the immunohistochemical study according to the manufacturers’ guidelines.

### 4.3. Visual Analysis of IHC Staining

The PD-L1 IHC staining was evaluated independently by 2 pathologists (P.P.A. and S.H.M.). The intensity of staining was assessed on a scale of 0 to 3 (0 = negative; 1 = mild; 2 = moderate; 3 = strong), while the extent of staining was assessed as a percentage of positive tumor cells or immune cells in increments of 10 percentage points. The positive expression of PD-1 and PD-L1 were defined as staining in ≥1% (partial or complete membranous pattern) of tumor cells in agreement with FDA-approved criteria [39]. A score incorporating both intensity and positivity (“H-score”) was calculated by multiplying staining intensity by percentage of positive tumor cells. Discrepant cases were reviewed to obtain a consensus. Immune cells showing membranous (partial or complete) staining for CD3 and CD8 were evaluated independently by the 2 pathologists in a similar fashion to obtain an H-score. Extramammary Paget disease cases were evaluated for CD3, CD8, PD-1, and PD-L1 in immune infiltrate and PD-1 and PD-L1 in tumor cells by visual analysis. The MPD cases were evaluated for PD-L1 in immune infiltrate and tumor cells by visual analysis.

To facilitate interpretation of the IHC staining and to better ascertain the presence and/or localization of tumor cells, cytokeratin 7 immunostaining was used in the majority of samples to localize the neoplastic cells.

### 4.4. Automated Image Analysis of Immune Infiltrate and PD-1

Eighteen (86%) of the 21 cases of EMPD were subjected to evaluation of CD3, CD8, and PD-1 in the immune infiltrate by automated image analysis. Three cases were excluded from automated image analysis because of metastatic disease in a lymph node (*n* = 1), a scant immune infiltrate (*n* = 1), or a small biopsy specimen (*n* = 1). The slides chosen were scanned at 20× magnification (Aperio ScanScope AT Turbo; Leica Biosystems). With the help of image analysis software (Aperio ImageScope), three 1 mm^2^ squares were designated in the areas of highest density of immune cells positive for the IHC marker in question. The number of cells positive for the marker in each of the 3 squares was then counted using a modified nuclear algorithm, as described in our previous study [18]. For each tumor, the mean number of cells positive per square was then calculated to obtain a final number of positive cells per mm^2^ for each case.

### 4.5. Statistical Methods

Demographic and clinical-pathologic parameters were summarized for all patients and for subgroups of patients defined by PD-L1 expression in tumor cells and the tumor-associated lymphocytic infiltrate. Categorical variables were summarized by frequencies and percentages and assessed using either Fisher’s exact test or generalized Fisher’s exact test; continuous measures were summarized by mean, standard deviation, median, and range (minimum, maximum) and assessed using either Wilcoxon rank–sum exact test or Kruskal–Wallis exact test. Correlations between continuous measures were determined using Spearman correlation coefficient.

Overall survival and disease-specific survival were computed from the date of sample collection to the last known follow-up date. Patients alive at the last follow-up date were censored for overall survival, and patients who died of causes other than EMPD and those who were alive at the last follow-up date were censored for disease-specific survival. Time to metastasis was computed from the date of sample collection to the date of metastasis. Patients who did not experience metastasis were censored at the last follow-up date. Overall survival, disease-specific survival, and time to metastasis were estimated using the Kaplan–Meier method, and the log–rank test was used to assess differences between groups.

All statistical analyses were performed using SAS 9.3 for Windows (SAS Institute Inc., Cary, NC, USA). All statistical tests used a significance level of 5%. No adjustments for multiple testing were made.

## 5. Conclusions

In our series, three of 21 patients with EMPD had PD-L1+ tumor cells, and the associated immune infiltrate consisted of varying densities of CD3+, CD8+, PD-1+, and PD-L1+ cells. With the exception of vital status and CD3+, there was no significant association between these findings and clinical-pathologic parameters, including overall survival and time to metastasis.

## Figures and Tables

**Table 1:** Summary of demographic and clinical-pathologic findings in patients with extramammary Paget disease (EMPD) and mammary Paget disease (MPD).

EMPD (*n* = 21)		MPD (*n* = 10)	
Characteristic	Value	Characteristic	Value
Age, years		Age, years	
Mean	67.7	Mean	50.5
Median	67.8	Median	51.9
Min, max	48.0, 79.0	Min, max	22.9, 70.6
Sex, *n*		Tumor type in ipsilateral breast, *n*	
Male	11	IDC	6
Female	10	ILC	1
Anatomic site, *n*		DCIS only	1
Perianal region	5	LCIS only	0
Vulva	6	DCIS + LCIS	1
Scrotum	7	No invasive or intraductal tumor	1
Other	3	ER status, *n*	
History of other cancer, *n*		Positive	5
Present	10	Negative	4
Absent	11	Not known	1
Local recurrence, *n*		PR status, *n*	
None	13	Positive	4
Single	2	Negative	5
Multiple	5	Not known	1
Persistent disease	1	HER2/neu status, *n*	
Metastasis, *n*		Positive	5
Yes	3	Negative	3
No	18	Not known	2
Vital status at last follow-up, *n*		Vital status at last follow-up, *n*	
Dead	6	Dead	1
Alive	11	Alive	3
Lost to follow-up	4	Lost to follow-up	6
Overall survival, %	62	Overall survival, %	90
Disease-specific survival, %	81	Disease-specific survival, %	90

Abbreviations: IDC, infiltrating ductal carcinoma; ILC, infiltrating lobular carcinoma; DCIS, ductal carcinoma in situ; LCIS, lobular carcinoma in situ; ER, estrogen receptor; PR, progesterone receptor; HER2, Human epidermal growth factor receptor-2.

**Table 2:** Clinical-pathologic findings by patient in patients with extramammary Paget disease (EMPD).

Case	PD-L1 in Tumor Cells by Visual Analysis (H-Score)	Density of Marker-Positive Cells in Immune Infiltrate by Image Analysis, Positive Cells/mm^2^			PD-L1 in Immune Infiltrate by Visual Analysis (H-Score)	Anatomic Site	Associated Invasive Component	Site of Metastasis	Survival
		CD3	CD8	PD-1					
Intraepithelial disease without invasion									
1	0	1650.3	991.4	190.0	1	Vulva	Absent	NA	AWOD
2	15	1062.1	475.1	103.6	1	Perianal	Absent	NA	AWOD
3	0	678.1	255.0	185.9	2	Scrotum	Absent	NA	AWOD
4	0	2045.8	684.2	212.0	20	Vulva	Absent	NA	AWOD
5	0	908.1	509.2	32.4	0	Suprapubic skin	Absent	NA	Died of unrelated cause
6	0	1484.2	1195.8	177.0	1	Perianal	Absent	NA	AWOD
7	0	481.2	294.9	115.6	0	Axilla	Absent	NA	Died of unknown cause
8	0	288.5	121.7	40.2	0	Vulva	Absent	NA	Lost to follow-up
9	2	1039.2	395.6	277.5	2	Perianal	Absent	NA	AWD
10	0	2114.9	1190.5	167.3	60	Scrotum	Absent	NA	AWOD
11	0	492.7	169.6	57.1	2	Scrotum	Absent	NA	Died of unknown cause
12	0	261.7	187.8	82.6	1	Perianal	Absent	NA	Lost to follow-up
13	0	1970.9	1263.0	330.4	1	Scrotum	Absent	NA	Lost to follow-up
14	0	543.1	237.8	14.4	2	Scrotum	Absent	NA	AWD
Invasive disease without metastasis									
15	0	598.7	225.7	33.5	1	Perianal	Focal dermal invasion	NA	Lost to follow-up
16	0	No analysis	No analysis	No analysis	0	Nose tip	Concurrent invasive adenocarcinoma in dermis of possible eccrine origin	NA	AWOD
17	0	1136.4	484.8	49.0	0	Vulva, perianal, vaginal, ectocervix	Invasive poorly differentiated adenocarcinoma in urinary bladder consistent with origin from EMPD 11 years after initial diagnosis (CK7+, CK20− GCDFP15+)	NA	AWOD
18	0	1927.3	1207.0	251.0	2	Vulva	Dermal invasion present (depth <0.5 mm)	NA	AWD
*Invasive disease with metastasis*									
19	1	No analysis	No analysis	No analysis	1	Scrotum	Dermal and lymphovascular invasion	Lymph nodes, skin, soft tissue, peritoneum, liver, bone	DOD
20	0	No analysis	No analysis	No analysis	0	Scrotum	Dermal invasion and concurrent rectal adenocarcinoma (CK7+, CK20+, CDX2−)	Lymph nodes, liver	DOD
21	0	728.5	389.3	40.5	10	Vulva	Dermal invasion (depth 10 mm)	Lymph nodes, liver	DOD

Abbreviations: NA, not applicable; AWOD, alive without disease; AWD, alive with disease; DOD, died of disease; CK, cytokeratin; GCDFP 15, Gross cystic disease fluid protein 15.

## Supplementary Materials

The following are available online at https://www.mdpi.com/2072-6694/11/6/754/s1, Table S1: Correlation of PD-L1 expression with density and composition of tumor-associated immune infiltrates and clinical-pathologic parameters in EMPD, Table S2: Correlation of density of CD3+ tumor-associated immune infiltrate and clinical-pathologic parameters in EMPD, Table S3: Correlation of density of CD8+ tumor-associated immune infiltrate and clinical-pathologic parameters in EMPD, Table S4. Correlation of density of PD-1+ tumor-associated immune infiltrate and clinical-pathologic parameters in EMPD, Figure S1. 1A–D: Representative sections of PD-L1 control. PD-L1 22C3-positive control of cell line NCL-H226 (A: low power and B: high power) and negative control: Cell line MCF-7 (C: low power and D: high power).
